# Perceptions on Medical Professionalism Among Future Healthcare Professionals: A Mixed Method Study

**DOI:** 10.7759/cureus.57573

**Published:** 2024-04-03

**Authors:** Vijaya Ramanathan, Punita Pushpanathan, Trupti Bodhare, Samir Bele

**Affiliations:** 1 Anatomy, All India Institute of Medical Sciences, Madurai, IND; 2 Anatomy, Meenakshi Medical College Hospital & Research Institute, Meenakshi Academy of Higher Education & Research (Deemed to be University), Kanchipuram, IND; 3 Physiology, Meenakshi Medical College Hospital & Research Institute, Meenakshi Academy of Higher Education & Research (Deemed to be University), Kanchipuram, IND; 4 Community & Family Medicine, All India Institute of Medical Sciences, Madurai, IND; 5 Community Medicine, Velammal Medical College Hospital & Research Institute, Madurai, IND

**Keywords:** mixed method study, assessment, teaching methods, undergraduate nursing students, undergraduate medical students, professional behavior, medical professionalism

## Abstract

Background

Accreditation councils across the world constantly examine policies and practices in professionalism in their medical curriculum. The National Medical Commission (NMC) in India has recognized the pressing need to reform and include professionalism in its undergraduate curriculum.

Objectives

The objective of this study was to explore the perspectives of medical and nursing students on professional behavior, suitable teaching-learning methods, and assessment strategies for curriculum integration.

Methodology

The study utilized a concurrent triangulation mixed method design, collecting both qualitative and quantitative data simultaneously to gain a comprehensive understanding of medical and nursing students' perceptions of professionalism. It included 83 final-year undergraduate medical students and 42 final-year undergraduate nursing students. The approval of the Institutional Review Board of Meenakshi Medical College Hospital & Research Institute was obtained. A semi-structured questionnaire consisting of demographic characteristics and opinions regarding academic professional behavior, teaching-learning, and evaluation of professionalism was used. Perceptions regarding the meaning of professionalism, behavior of professionalism to be emphasized in teaching, and pressing challenges of professionalism were explored. Students were also asked to rank the best behavior associated with professionalism. Frequency and percentages were used for descriptive statistics. Means and standard deviations were calculated for continuous variables. An unpaired t-test was used to determine a statistically significant difference between the means in the two groups. The quantitative data was analyzed with R programming and content analysis was performed for the qualitative data using ATLAS.ti qualitative data analysis software.

Results

Unexplained/unauthorized absence from academic activities (2.39 ± 1.553), not following the timeline (2.41 ± 1.560), making fun of patients and peers (2.16 ± 1.619), cheating in the exams (2.37 ± 1.651), and inebriation (2.39 ± 1.666) were unacceptable behaviors by undergraduate medical students compared with nursing students. Clinical experience (1.54 ± 0.857) and role models (1.74 ± 0.935) were the highly acceptable methods of teaching professionalism and interprofessional interactions (1.58 ± 0.650) and awards (1.98 ± 1.100) were the most common suggestions to improve the course curriculum by both groups. Community/field activity (1.78 ± 0.860) and clinical examination (1.89 ±1.123) were the most preferred methods of evaluation of professionalism. According to the students, dedication, honesty, respect, and self-improvement were identified as the best behaviors associated with professionalism.

Conclusions

The study revealed that students had a wide range of perspectives about professionalism. Different unprofessional acts were acceptable to students. The causes of these perspectives need to be explored and resolved to promote professionalism. Students identified the need for strong positive role models and frequent clinical experiences, along with improved interprofessional interactions and awards to improve teaching and learning professionalism. Community/field activity was the preferred assessment method proposed by the students. Medical institutions must promote these components in curriculum, faculty development, and clinical settings to foster the development of professionalism among students.

## Introduction

Medical students learn the attributes of professionalism from their teachers and exhibit it in their careers. Professionalism was a part of the hidden curriculum but now has surfaced and needs to be emphasized explicitly. The Accreditation Council for Graduate Medical Education (ACGME), Canadian Medical Education Directions for Specialists (CANMEDS), and General Medical Council (GMC) constantly examine policies and practices regarding professionalism and ethics in their medical curriculum [1]. The NMC in India has recognized the pressing need to reform and include professionalism in its undergraduate curriculum. In 2019, the NMC introduced a competency-based undergraduate curriculum. The updated curriculum integrates teaching and learning Attitude, Ethics, and Communication Skills (AETCOM) from the first year of training throughout the entire duration, aiming to improve the practices and enhance the reputation of medical professionals [2].

Medical and paramedical healthcare professionals are entrusted with the health and well-being of people. Therefore, medical education should prioritize teaching individuals who adhere to timeless principles like respect for others, empathy, compassion, honesty, integrity, altruism, and professional excellence. These traits contribute to establishing a robust connection between healthcare professionals and society. Students are trained well in knowledge, skills, and clinical acumen and are periodically assessed in these competencies but they demonstrate reduced professional conduct. Furthermore, there are no reliable tools to detect low levels of professionalism, follow-up is difficult, and progress in professionalism is difficult to track. Professionalism is the sum of values, conducts, and relationships exhibited by physicians in public to guarantee better health services. Professionalism is a complex concept and it is difficult to know how and when to introduce professionalism in medical school curriculum [3].

Students act more professionally in the beginning of medical education and introducing professionalism early in their curriculum is advisable to reap maximum benefit. Participants support the importance of training for professionalism and the value of its relevance in practicing medicine is pertinent. With the advent of commercialism in the medical profession, healthcare professionals are caught between patients and proﬁt makers. The need to maintain professionalism has increased. It solely depends on healthcare professionals as opposed to the policymakers, politicians, and administrators. There is no single best teaching method, a blend of methods like visual aids, small group discussions, role-playing, and incorporation into problem-based learning is essential. Various assessment methods are suggested including faculty observation of students, portfolios, peer and self-assessment, objective structured clinical examinations (OSCE), objective structured practical examinations (OSPE), written examinations, and standardized patients to assess professionalism [4,5].

The student's response is necessary to assess a program. These responses constitute the first level of Kirkpatrick's evaluation model [5]. The students' perspectives and experiences are significant, yet there is minimal data on their response to professionalism in India.

The study aims to explore the perspectives of medical and nursing students on professional behavior, suitable teaching-learning methods, and assessment strategies for curriculum integration. Studies on overall perspectives about professionalism from both medical and paramedical students are available worldwide. However, there are limited studies that find an association between various aspects of professionalism and how the medical and paramedical students differ in their understanding, and the importance they give to this aspect of core competency is concentrated in this study.

## Materials and methods

Study design and setting

The study utilized a concurrent triangulation mixed method design, collecting both qualitative and quantitative data simultaneously to gain a comprehensive understanding of medical and nursing students' perceptions of professionalism. The study was conducted in Meenakshi Medical College Hospital & Research Institute, Kanchipuram, Tamil Nadu.

Participants

The two groups of students, consisting of final-year undergraduate medical students and undergraduate nursing students, were selected through convenient sampling. The data was collected over a period of three months.

Inclusion and exclusion criteria

The final-year undergraduate medical and nursing students who were willing to take part in the research were included in the study, whereas the students who were not willing to take part in the study were not included in the study.

Ethical considerations

The approval of the Institutional Review Board of Meenakshi Medical College Hospital & Research Institute was obtained before the start of the study. Written informed consent was obtained from the students. Strict data confidentiality was maintained throughout the research. 

Parameters measured

Quantitative Measures

A semi-structured questionnaire was developed after conducting an extensive review of the literature on relevant studies in the field. Expert faculties from the Medical Education Unit of the same institution conducted the validation. The questionnaire was used to collect basic demographic details like age, gender, residence, year of study, etc. The participant inputs on the activities related to academic professionalism were measured in the domain of lack of involvement, in-disciplined behavior, and dishonest behavior. The questions about teaching and learning professionalism include the best teaching methodology, effective components in teaching professionalism, and potential improvements in the curriculum for incorporating professionalism. The questions regarding the assessment of professionalism include the best assessment method and frequency of assessment of professionalism. A five-point Likert scale was used with options ranging from (one- strongly agree to five- strongly disagree). Students were also asked to rank the best behavior associated with professionalism in order from one to ten in areas like compassion, dedication, altruism, empathy, etc.

Qualitative Measures

To explore a deeper understanding, the students were asked to write responses to open-ended questions on what professionalism means to them, which behavior of professionalism needs to be emphasized most in their education, and the most pressing challenges of professionalism students are expected to face in their careers.

Pilot study

A pilot study was conducted among a group of 20 students (seven undergraduate nursing students and 13 undergraduate medical students) to evaluate the readability, comprehensibility, and graphical order of the instrument and to ascertain that participants comprehended the items as intended. Pilot samples represented the intended research population and used the same inclusion and exclusion criteria as the original research. Reliability of the questionnaire was assessed using Cronbach's alpha, which yielded a value greater than 0.7. The participants of the pilot study were excluded from the sample of the main study.

Statistical analysis

Frequency and percentages were used for descriptive statistics. Means and standard deviations were calculated for continuous variables. An unpaired t-test was used to determine a statistically significant difference between the means in the two groups. A p-value of ≤0.05 was used as the threshold for statistical significance. For the qualitative component, an inductive approach was used, using a conventional content analysis of the students' written responses. The quantitative data was analyzed with R programming and content analysis was performed for the qualitative data using ATLAS.ti qualitative data analysis software.

## Results

In the study, a total of 83 final-year MBBS students and 42 final-year BSc nursing students participated. Table 1 shows the participant's gender, age, and residence. Participants had a mean age of 21.29 ± 0.693, with 88 (70.4%) being female and 98 (78.4%) belonging to urban areas.

**Table 1:** Demographic characteristics of the participants

S. No.	Variables		Medical Students n (%)	Nursing Students n (%)	Total n (%)
1	Gender	Male	30 (36.1%)	7 (16.7%)	37 (29.6%)
		Female	53 (63.9%)	35 (83.3%)	88 (70.4 %)
2	Residence	Rural	4 (4.8%)	23 (54.8%)	27 (21.6%)
		Urban	79 (95.2%)	19 (45.2%)	98 (78.4%)
3	Age (Mean ± SD)		21.27 ± 0.646	21.33 ± 0.786	21.29 ± 0.693

Table 2 reflects the participants' views on breaches of academic professionalism on three subscales. The lowest scores are obtained in the domain of lack of engagement (2.67 ± 1.30) followed by disrespectful behavior (2.82 ± 1.35) and dishonest behavior (2.80 ± 1.32).

**Table 2:** Participants' views on breaches of academic professionalism *Statistically significant (p≤0.05)

S. No.	Questions	Medical Students (Mean ± SD)	Nursing Students (Mean ± SD)	Total (Mean ± SD)	p-value
	Lack of Engagement	2.44 ± 1.34	3.13 ± 1.07	2.67 ± 1.30	0.215
1	Do not follow the timeline	2.41 ± 1.560	3.17 ± 1.447	2.66 ± 1.56	0.694
2	Unexplained/unauthorized absence from academic activities	2.39 ± 1.553	3.36 ± 1.445	2.71 ± 1.58	0.348
3	Not participating in co-curricular/ extra-curricular activities	2.88 ± 1.434	2.88 ± 1.517	2.65 ± 1.47	0.378
Disrespectful behavior		2.44 ± 1.40	3.55 ± 0.88	2.82 ± 1.35	0.256
4	Inappropriate use of social media	2.66 ± 1.508	3.36 ± 1.376	2.90 ± 1.496	0.267
5	Making fun of patients, peers or physicians	2.16 ± 1.619	3.31 ± 1.316	2.54 ± 1.614	0.115
6	Poor condition of white coats	2.36 ± 1.558	3.69 ± 1.297	2.81 ± 1.600	0.077
7	Wearing untidy dress	2.34 ± 1.655	3.60 ± 1.345	2.76 ± 1.663	0.064
8	Not maintaining professional appearance and attire	2.45 ± 1.698	3.81 ± 1.131	2.61 ± 1.658	*0.000
9	Wear white coats/scrubs in the non-clinical environment	2.70 ± 1.454	3.55 ± 1.214	2.98 ± 1.431	0.105
Dishonest behavior		2.42± 1,33	3.55 ± 0.91	2.80 ± 1.32	0.618
10	Using mobile/laptop/electronic devices during class	2.33 ± 1.449	3.31 ± 1.405	2.66 ± 1.503	0.766
11	Giving proxy/signing another student in a class	2.47 ± 1.310	3.24 ± 1.503	2.73 ± 1.328	0.851
12	Cheating in the exams to obtain more marks	2.37 ± 1.651	3.67 ± 1.300	2.81 ± 1.654	*0.010
13	Eating in front of patients/in patient corridors	2.43 ± 1.500	3.90 ± 1.078	2.93± 1.535	*0.005
14	Accepting gifts from pharmaceutical representative	2.59 ± 1.514	3.38 ± 1.361	2.86 ± 1.506	0.200
15	Inebriation/consuming alcohol during medical college events	2.39 ± 1.666	3.83 ± 1.286	2.57 ± 1.690	*0.003

In the domain of lack of engagement, unexplained/unauthorized absence from academic activities (2.39 ± 1.553) and not following the timeline (2.41 ± 1.560) were unacceptable professional behavior by MBBS students.

In the domain of disrespectful behavior, making fun of patients and peers was unacceptable by MBBS students (2.16 ± 1.619), not maintaining a professional appearance or attire was unacceptable by MBBS students (2.45 ± 1.698) whereas it was acceptable behavior by nursing students (3.81 ± 1.131). This difference was statistically significant (p=0.000).

In the domain of dishonest behaviors, cheating in the exams to obtain more marks (2.37 ± 1.651), eating in front of the patient (2.43 ± 1.500), and inebriation (2.39 ± 1.666) were unacceptable behavior among MBBS students compared with nursing students and this difference was statistically significant (p< 0.05).

Table 3 depicts the views of effective components in a professionalism curriculum and suggestions to improve the curriculum. In both the groups, clinical experience (1.54 ± 0.857) and role models (1.74 ± 0.935) were the highly acceptable methods of teaching professionalism, whereas online classes (2.82 ± 1.272) and tutorials (2.74 ± 1.199) were the least preferred methods of learning professionalism in both the groups.

**Table 3:** Participants' views on successful professionalism course in teaching professionalism *Statistically significant (p≤0.05)

S. No.	Questions	Medical Students (Mean ± SD)	Nursing Student (Mean ± SD)	Total (Mean ± SD)	p-value
1	Lectures	2.48 ± 1.233	1.52 ± 0.707	2.16 ± 1.174	*0.001
2	Online classes	2.84 ± 1.292	2.76 ± 1.246	2.82 ± 1.272	0.955
3	Tutorials	2.83 ± 1.146	2.55 ± 1.292	2.74 ± 1.199	0.166
4	Clinical experience	1.49 ± 0.861	1.64 ± 0.850	1.54 ± 0.857	0.621
5	Role play	2.39 ± 1.146	2.05 ± 0.936	2.27 ± 1.088	*0.032
6	Simulation	2.47 ± 1.016	2.40 ± 1.061	2.45 ± 1.027	0.542
7	Apprenticeship	2.34 ± 1.107	2.60 ± 1.083	2.42 ± 1.102	0.911
8	Role models	1.73 ± 0.951	1.74 ± 0.912	1.74 ± 0.935	0.299
9	Interprofessional interactions	1.48 ± 0.549	1.79 ± 0.782	1.58 ± 0.650	0.545
10	Guest lectures	2.27 ± 0.964	2.10 ± 0.878	2.21 ± 0.936	0.186
11	Awards to staff and students	1.95 ± 1.047	2.05 ± 1.209	1.98 ± 1.100	0.381
12	Publication of disciplinary measures	2.14 ± 1.014	1.98 ± 0.841	2.09 ± 0.959	0.111
13	Special interest group on professionalism / having a separate team.	2.05 ± 1.047	2.26 ± 1.083	2.12 ± 1.060	0.718

Interprofessional interactions (1.58 ± 0.650) and awards to staff and students (1.98 ± 1.100) were the most common suggestions to improve the course curriculum by both groups. There was no statistically significant difference in the responses by both the groups (p>0.05).

Table 4 reveals the perceptions of the students regarding various methods of assessment and frequency of assessment of professionalism. Essay writing (2.90 ± 1.300) and multiple-choice questions (2.42 ± 1.309) were the least preferred method for evaluation of professionalism, whereas community/field activity (1.78 ± 0.860) and clinical examination (1.89 ± 1.123) were the most preferred method of evaluation of professionalism in both the groups.

**Table 4:** Participants' perceptions regarding the assessment of professionalism *Statistically significant (p≤0.05)

S. No.	Questions	Medical Students (Mean ± SD)	Nursing Students (Mean ± SD)	Total (Mean ± SD)	p-value
Methods of assessment of professionalism					
1	Essays	3.01 ± 1.274	2.67 ± 1.337	2.90 ± 1.300	0.477
2	Multiple choice questions	2.81 ± 1.374	1.67 ± 0.721	2.42 ± 1.309	*0.000
3	Project Work	2.47 ± 1.097	1.90 ± 0.906	2.28 ± 1.067	*0.019
4	Community/Field Activity	1.65 ± 0.788	2.02 ± 0.950	1.78 ± 0.860	0.902
5	Roleplay	2.31 ± 1.239	2.12 ± 1.131	2.25 ± 1.203	0.122
6	Clinical Examination	1.77 ± 1.051	2.12 ± 1.234	1.89 ± 1.123	0.368
7	OSPE/OSCE	2.52 ± 1.108	2.19 ± 0.994	2.41 ± 1.078	0.258
8	Assignments	3.08 ± 1.232	2.12 ± 1.273	2.76 ± 1.322	0.967
9	Workplace-based assessment	1.84 ± 1.006	2.02 ± 0.869	1.90 ± 0.962	0.523
10	Peer (Friends/batch mates/ colleague) assessment	1.90 ± 1.111	2.00 ± 1.012	1.94 ± 1.076	0.255
Frequency of assessment					
11	At the end of every year	2.05 ± 0.840	2.00 ± 0.541	2.03 ± 0.751	*0.057
12	At the beginning of final year	1.90 ± 1.408	1.90 ± 1.055	2.45 ± 1.353	*0.002
13	At the end of final year	1.64 ± 1.204	1.64 ± 0.932	1.86 ± 1.127	0.720

Multiple choice questions (2.81 ± 1.374) and project work (2.28 ± 1.067) were somewhat acceptable by the MBBS students, whereas multiple choice questions (1.67 ± 0.721) and project work (1.90 ± 0.906) were highly acceptable methods of assessment in nursing students and this group difference was statistically significant (p<0.05).

Regarding frequency of assessment of professionalism, both the groups agreed that it should be evaluated at the end of the final year.

Table 5 presents the opinion of the students for 10 different behaviors across various ranks along with their corresponding frequencies and percentages. The table reveals how individuals order ten behaviors across different ranks. Particularly, dedication is predominantly ranked at 1, representing 30 (24%) of responses, suggesting its paramount importance. Honesty is placed at rank 2, representing 26 (20.8%) of responses. Respect is often placed at rank 3, representing 28 (22.4%) of responses. self-improvement is frequently linked with rank 4, comprising 16 (12.8%) of responses. Integrity is often placed at rank 9, representing 24 (19.2%) of responses. Accountability is mainly ranked at 10, accounting for 28 (22.4%) of responses and altruism/unselfishness garners the highest frequency at rank 10, with 30 (24%) of responses, indicating its perceived association with lower ranks.

**Table 5:** Participants' views about the best behavior associated with professionalism #1 being the best behavior and 10 being the least best behavior associated with professionalism

#Rank	Compassion n (%)	Dedication n (%)	Altruism/ unselfishness n (%)	Empathy n (%)	Respect n (%)	Self-Improvement n (%)	Honesty n (%)	Accountability n (%)	Integrity n (%)	Responsibility n (%)
1	5 (4%)	30 (24%)	5 (4%)	8 (6.4%)	15 (12%)	14 (11.2%)	20 (16%)	3 (2.4%)	5 (4%)	14 (11.2%)
2	12 (9.6%)	8 (6.4%)	5 (4%)	7 (5.6%)	23 (18.4%)	12 (9.6%)	26 (20.8%)	2 (1.6%)	8 (6.4%)	20 (16%)
3	4 (3.2%)	20 (16%)	3 (2.4%)	7 (5.6%)	28 (22.4%)	9 (7.2%)	15 (12%)	10 (8%)	9 (7.2%)	22 (17.6%)
4	11 (8.8%)	10 (8%)	13 (10.4%)	13 (10.4%)	18 (14.4%)	16 (12.8%)	15 (12%)	5 (4%)	9 (7.2%)	15 (12%)
5	13 (10.4%)	8 (6.4%)	17 (13.6%)	22 (17.6%)	12 (9.6%)	13 (10.4%)	12 (9.6%)	11 (8.8%)	7 (5.6%)	10 (8%)
6	16 (12.8%)	8 (6.4%)	8 (6.4%)	19 (15.2%)	12 (9.6%)	12 (9.6%)	12 (9.6%)	15 (12%)	11 (8.8%)	10 (8%)
7	22 (17.6%)	12 (9.6%)	14 (11.2%)	9 (7.2%)	5 (4%)	13 (10.4%)	8 (6.4%)	12 (9.6%)	20 (16%)	11 (8.8%)
8	14 (11.2%)	12 (9.6%)	15 (12%)	13 (10.4%)	6 (4.8%)	15 (12%)	3 (2.4%)	20 (16%)	22 (17.6%)	6 (4.8%)
9	13 (10.4%)	13 (10.4%)	15 (12%)	13 (10.4%)	2 (1.6%)	12 (9.6%)	10 (8%)	19 (15.2%)	24 (19.2%)	6 (4.8%)
10	15 (12%)	4 (3.2%)	30 (24%)	14 (11.2%)	4 (3.2%)	9 (7.2%)	4 (3.2%)	28 (22.4%)	10 (8%)	11 (8.8%)

Qualitative analysis

The data is organized into codes, and sub-categories within the three main categories: (a) What does professionalism mean to you? (b) Which behavior of professionalism needs to be emphasized at this point in your education? (c) What is the most pressing challenge to professionalism you expect to face in your career? 

What Does Professionalism Mean to You?

Participants described how they viewed professionalism. Professionalism was seen as a combination of learned skills, ethical conduct, and dedication to patient care. The various subcategories obtained were ethical patient care, continuous self-improvement, trust and responsibility, equality and respect, commitment to service, and pride and passion. Around 24 participants believe that professionalism is upholding ethical standards in patient treatment. “It means doing justice to your profession without breaking ethics or law” (p. 13). “It is the ability to maintain medical ethics and etiquette in all aspects of medical practice and not to corrupt medicine” (p. 17). Eleven participants stated that professionalism is a commitment to ongoing learning and professional growth. "Professionalism means a complete dedication towards what we do with honesty. Always improving themselves with the recent advances and events regarding the profession." (p. 31).

Which Behavior of Professionalism Needs to be Emphasized at This Point in Your Education? 

Empathy and compassion, ethical integrity, ethical conduct, effective communication, dedication and responsibility, and respectful interaction are the various aspects of professionalism that students believe should be emphasized in their education. Around 15 participants recognize the significance of being dedicated to their profession and taking responsibility for patient care. “Respect, dedication, compassion, unselfishness must be emphasized" (p. 36). Nine participants stated that focusing on developing strong communication skills to facilitate rapport with patients, colleagues, and faculty shall be emphasized. "Communication with the patient and punctuality" (p.58).

What Is the Most Pressing Challenge to Professionalism You Expect to Face in Your Career? 

The various challenges that the participants anticipate facing in their careers regarding professionalism include personal and professional balance, delivering patient-centered care, ethical practice and accountability, continuous learning and improvement, financial and workplace challenges, peer pressure, and professional environment. Additionally managing time effectively, facing patients and their diverse needs, navigating internet misinformation affecting patient perceptions, managing uncooperative or aggressive patients, and handling emergencies with composure were the pressing concerns. Eight participants expressed the challenges of delivering patient-centered care. “Lack of faith from patients because of improper information from the internet, judging a doctor always, always being put into scrutiny and comparison” (p. 59). “The ill-informed patients who surf the internet to show that they have the required knowledge” (p. 14). Few students expressed concerns regarding emotional resilience which is coping with stress, fear, and anxiety in healthcare settings. “Critics, abuse of medical profession the only fear of everyone in the future” (p.10).

## Discussion

This research presents empirical evidence about the perspectives of undergraduate medical students and nursing students on the concept of professionalism.

In the context of acceptable and unacceptable professional behaviors, we obtained the lowest score in the domain of lack of engagement, such as arriving late or missing classes or other assigned activities, and not participating in curricular and extracurricular activities. The cause of this may be ascribed to inadequate motivation [6]. Signs of disengagement may manifest as a prelude to avoiding interactions with patients, failing to contribute to patient care, leaving the hospital during a shift, and demonstrating poor levels of involvement. Another motif enquired was disrespectful behavior. We observed inappropriate body language, attire, and making fun of patients and peers were highly ranked unacceptable behaviors among the study participants. Disrespectful behavior by students may impact interactions with instructors, staff, patients, and peers. Dishonest behaviors cover student integrity issues This includes cheating, lying, inebriation, and breaching norms and regulations. Instances of dishonesty occurred in the classroom via the act of falsifying signatures, providing fake reasons for absence enlisting a peer to sign in on an attendance roster, or soliciting other students to complete for other students. These behaviors may damage patient confidence, professional standards, and conflicts of interest. Our findings are consistent with the findings of a study conducted by Kulac et al. In his study, more than 90% of medical students reported that they had witnessed absence from mandatory lectures, wearing white coats/scrubs in a nonclinical environment or out of the hospital, and frequent inebriation at school events. Similarly, Reddy et al. reported a noticeable rise in the occurrence of unprofessional acts like late to rounds, absent from mandatory lectures, use of workrooms for nonclinical activities, and taking food meant for patients among students during their clerkships. The author concluded that engaging in unprofessional acts was linked to seeing these actions as acceptable [7,8]. Our results also correlate with the research conducted by Hejri et al., who identified the most often reported form of academic dishonesty as 'impersonating an absent student in a class'. Shukr et al. did research at two medical institutions in Pakistan, which yielded comparable results. The most prevalent forms of dishonest activity reported were proxy attendance cheating in examinations, and completing assignments for other students [9,10].

Response from students indicates that clinical experience and role modeling are the most crucial aspects of the educational experience for developing professionalism. The findings of our study align with the research conducted by Byszewski et al. and Dhikale et al., which indicated that students favored faculty role modeling and case-based discussions as effective methods for acquiring professionalism [1,11]. The students' perspectives of the most compelling suggestion on how to enhance the curriculum was to improve inter-professional contacts and provide awards to staff and students. [1] Considering the students' input can enhance the curriculum and instill a sense of professionalism in teaching.

Community/field activity and clinical examination were the most preferred methods of evaluation of professionalism in both groups. Students felt evaluation in the form of essay writing and multiple-choice questions was not required. Evaluating professionalism is a recognized challenge in developing a comprehensive professionalism program. The author's conclusion emphasizes the creation of effective qualitative methods. Additionally, the author highlights the need for deeper investigation into the assessment environment [12]. A systematic review done by de Mendonça showed a significant improvement in communication with patients, health education, respect for the interdisciplinary team, confidentiality, and attitudes like compassion and respect for diversity following the interventions and field experiences [13].

Regarding best behavior associated with professionalism, we found that dedication is predominantly ranked at 1, honesty is associated with rank 2, respect at rank 3, and self-improvement is placed at rank 4. The results of our research align with the findings of the study done by Byszewski et al. The three highest-ranked values obtained by the author were respect, integrity, and honesty. In a Kenyan teaching hospital, Ojuka et al. examined how doctors, students, and patients perceive professionalism. Respect was the most commonly expressed professionalism component in the interview along with service quality, and patient care [14]. This is consistent with the findings of our study.

The participants shared their perspectives on what constitutes professionalism and their views predominately included ethical patient care, continuous self-improvement, trust and responsibility, equality and respect, commitment to service, and pride and passion. Kirk et al. defined professionalism as prioritizing patient care, autonomy, and social justice. He stressed the importance of considering the patient's values and preferences and aligning them with the physician's clinical judgment [15].

In our study, professional challenges participants expect to face include personal and professional balance, patient-centered care, ethical practice and accountability, continuous learning and improvement, financial and workplace challenges, peer pressure, and a professional environment. Kumar et al. studied the challenges to professionalism and ethics in perioperative clinical practice and demonstrated that workplace pressures, conflicts, ego, prescribed rules and procedures, the need to meet healthcare targets, desire for autonomy, and multidisciplinary patient care can make professionalism and ethics difficult to thrive which are similar to the findings of our study [16].

Limitations

The results of our research are limited to the perspectives of students from a single medical and nursing college. Hence, the finding may lack generalizability.

## Conclusions

The study revealed that students had a wide range of perspectives about suitable professional behavior. Different unprofessional acts were acceptable to students. It is necessary to explore and address the reasons behind these perspectives to enhance professionalism. Traditional teaching and assessment methods were not favored by the students. Students identify the need for strong positive role models and frequent clinical experiences, along with improved interprofessional interactions and awards to improve teaching and learning professionalism. Community/field activity was the preferred assessment method proposed by the students. Medical institutions must promote these components in curriculum, faculty development, and clinical settings to foster the development of professionalism among students. 

## References

[1] Byszewski A, Hendelman W, McGuinty C, Moineau G (2012). Wanted: role models--medical students' perceptions of professionalism. BMC Med Educ.

[2] Desai MK, Kapadia JD (2022). Medical professionalism and ethics. J Pharmacol Pharmacother.

[3] Goldie J (2013). Assessment of professionalism: a consolidation of current thinking. Med Teach.

[4] Altirkawi K (2014). Teaching professionalism in medicine: what, why and how?. Sudan J Paediatr.

[5] Shumway JM, Harden RM (2003). AMEE Guide No. 25: The assessment of learning outcomes for the competent and reflective physician. Med Teach.

[6] Ten Cate TJ, Kusurkar RA, Williams GC (2011). How self-determination theory can assist our understanding of the teaching and learning processes in medical education. AMEE guide No. 59. Med Teach.

[7] Kulac E, Sezik M, Asci H, Doguc DK (2013). Medical students' participation in and perception of unprofessional behaviors: comparison of preclinical and clinical phases. Adv Physiol Educ.

[8] Reddy ST, Farnan JM, Yoon JD, Leo T, Upadhyay GA, Humphrey HJ, Arora VM (2007). Third-year medical students' participation in and perceptions of unprofessional behaviors. Acad Med.

[9] Hejri SM, Zendehdel K, Asghari F, Fotouhi A, Rashidian A (2013). Academic disintegrity among medical students: a randomised response technique study. Med Educ.

[10] Shukr I, Roff S (2015). Prevalence of lapses in academic integrity in two Pakistani medical colleges. Med Teach.

[11] Dhikale PT, Shrivastava SR, Srinivasan S (2020). Perspectives about professionalism among undergraduate students in a medical college in India: a qualitative study. Indian J Community Med.

[12] Arnold EL, Blank LL, Race KE, Cipparrone N (1998). Can professionalism be measured? The development of a scale for use in the medical environment. Acad Med.

[13] de Mendonça ET, Cotta RM, Lelis VP, Carvalho Junior PM (2016). Assessment of professionalism in students of health-related courses: a systematic review. Comunicação Saúde Educação.

[14] Ojuka DK, Olenja JM, Mwango'mbe NJ, Yang EB, Macleod JB (2016). Perception of medical professionalism among the surgical community in the University of Nairobi: a mixed method study. BMC Med Educ.

[15] Kirk LM (2007). Professionalism in medicine: definitions and considerations for teaching. Proc (Bayl Univ Med Cent).

[16] Kumar B (2021). Challenges to professionalism and ethics in perioperative clinical practice. Br J Hosp Med (Lond).

